# The DNA-helicase HELLS drives ALK^−^ ALCL proliferation by the transcriptional control of a cytokinesis-related program

**DOI:** 10.1038/s41419-021-03425-0

**Published:** 2021-01-27

**Authors:** Annalisa Tameni, Elisabetta Sauta, Valentina Mularoni, Federica Torricelli, Gloria Manzotti, Giorgio Inghirami, Riccardo Bellazzi, Valentina Fragliasso, Alessia Ciarrocchi

**Affiliations:** 1 Clinical and Experimental Medicine PhD Program, University of Modena and Reggio Emilia, Modena, 41125 Italy; 2 Laboratory of Translational Research, Azienda USL-IRCCS di Reggio Emilia, Reggio Emilia, 42123 Italy; 3 Department of Electrical, Computer and Biomedical Engineering, University of Pavia, Via Ferrata 5, 27100 Pavia, Italy; 4 Department of Pathology and Laboratory Medicine, Weill Cornell Medicine, New York, NY 10065 USA

**Keywords:** Cytokinesis, Transcriptional regulatory elements

## Abstract

Deregulation of chromatin modifiers, including DNA helicases, is emerging as one of the mechanisms underlying the transformation of anaplastic lymphoma kinase negative (ALK^−^) anaplastic large cell lymphoma (ALCL). We recently identified the DNA-helicase HELLS as central for proficient ALK^−^ALCL proliferation and progression. Here we assessed in detail its function by performing RNA-sequencing profiling coupled with bioinformatic prediction to identify HELLS targets and transcriptional cooperators. We demonstrated that HELLS, together with the transcription factor YY1, contributes to an appropriate cytokinesis via the transcriptional regulation of genes involved in cleavage furrow regulation. Binding target promoters, HELLS primes YY1 recruitment and transcriptional activation of cytoskeleton genes including the small GTPases RhoA and RhoU and their effector kinase Pak2. Single or multiple knockdowns of these genes reveal that RhoA and RhoU mediate HELLS effects on cell proliferation and cell division of ALK^−^ALCLs. Collectively, our work demonstrates the transcriptional role of HELLS in orchestrating a complex transcriptional program sustaining neoplastic features of ALK^−^ALCL.

## Introduction

Anaplastic large cell lymphomas (ALCLs) are a group of neoplasms arising from the transformation of mature T-cell^1^. The presence of chromosomal rearrangements involving the *ALK* gene stratifies ALCLs in ALK^+^ and ALK^−^ identifying two distinct diseases with different clinical behavior and prognosis^2–4^. ALK^−^ are known to be the most aggressive subtype of ALCL and the life expectancy of affected patients is significantly reduced by the lack of effective therapies^4–6^. The molecular bases of ALK^−^ALCLs remain largely unknown as a consequence of the biological complexity of this disease and of its relative rarity that reduces the possibility of extensive profiling^7–11^. Understanding the mechanisms that underline the development and evolution of ALK^−^ALCLs is crucial to define the molecular vulnerabilities of these lymphomas and to develop specific therapeutic strategies.

DNA helicases are a class of enzymes whose primary function is to unpack DNA. Considered as molecular motors, these proteins unwind the DNA exploiting ATP hydrolysis, thus facilitating replication and transcription^12–14^. For their importance in DNA maintenance, repair, and chromosomal segregation, helicases are considered guardian of the genomic stability^15,16^. Thus, it is not surprising that genetic or transcriptional alterations in many members of this family have been linked to different disease conditions including predisposition to cancer^17–20^. Besides, the relevance of these enzymes in promoting transcription initiation and cancer progression, has recently started to emerge as essential mechanism to explain their contribution to cell biology^17,21,22^. In virtue of their centrality in this fundamental mechanism, helicases are currently counted among the most appealing targets for cancer therapies.

We recently demonstrated that HELLS, a DNA helicase of the SWI/SNF2 family, is required for proficient ALK^−^ALCLs proliferation. We showed that HELLS is a downstream target of STAT3 and that its expression is controlled by the ALK^−^ALCLs specific lncRNA BlackMamba. Besides, we demonstrated that BlackMamba interacts with HELLS driving its positioning on target promoters, suggesting that the role of this helicase in this tumor setting may rely on its transcriptional activity^8^.

In this work, we explored in detail the molecular function of HELLS investigating the transcriptional program through which this helicase supports ALK^−^ALCLs. We provided evidence that HELLS coordinates the expression of a program of genes involved in cytoskeleton organization and cytokinesis thus orchestrating timing of cell division. We also showed that YY1 is a central partner of HELLS in supporting this program.

## Materials and methods

### Cell culture and treatments

The human ALK^−^ALCL cell line MAC2A was a kind gift of Dr. Giorgio Inghirami. The human Breast Implanted Associated (BIA)-ALCL cell line TLBR-2 was a kind gift of Dr. Alain Epstein. Cell identity was determined yearly. All cell lines were genotyped and routinely tested for *Mycoplasma* contamination. Cell lines were cultured in RPMI-1640 medium (Gibco) supplemented with 10% FBS at 37 °C in an atmosphere of 5% CO_2_. TLBR-2 cells were supplemented with IL2 (20U/ml).

Doxycycline hyclate was purchased from Sigma and dissolved in H2O.

### Cell growth and cell division

For cell growth assays, cells were washed with phosphate-buffered saline, seeded at 2.5×10^5^ cells/ml and treated with 100 nM doxycycline. Viable cells were counted by trypan blue exclusion.

### Plasmids and viral infections

pLKO Tet-On vectors expressing shRNAs against HELLS and lncRNA BlackMamba were generated by cloning synthetic double-stranded oligonucleotides into pLKO Tet-On vector (Addgene #21915). Vectors were packaged into lentiviral particles HEK 293T-cell line and used for infection of low passages MAC2A or TLBR-2 at multiplicity of infection. Cells were selected with 0.5 or 1 μg/ml of puromycin (MAC2A and TLBR-2 respectively) for 3 days.

The list of shRNAs sequences is provided in Supplementary Table 1.

### siRNA transfection

MAC2A and TLBR-2 cells (1×10^6) were transfected with 30 nM siRNA concentration for single KD. SiRNA transfections were performed using the Cell Line Nucleofector Kit SF and Amaxa 4D Nucleofector (program DS-130 for TLBR-2, FI115 for MAC2A). Twenty-four hours after transfection, cells were harvested and plated 2.5 × 105 cells/ml. For siRNA scramble, we used a Silencer Select negative control (Ambion, Life Technologies). For PAK2 and RHOA we used a Silencer Select Validated siRNAs, ID:s10022 and ID:s759, respectively (Ambion, Life Technologies). For RHOU we used two different Silencer Selected Pre-designed siRNAs ID:224502, ID: s33826 (Ambion, Life Technologies). For YY1, we used a Silencer Select Validated siRNAs: ID:s14958 **(**Ambion, Life Technologies**)**

### RNA extraction and quantitative PCR (qRT-PCR)

Total RNA was extracted by TRIzol (Thermo Fisher Scientific) according to the manufacture’s instructions. One microgram of total RNA was retrotranscribed using the iScript cDNA kit, (Biorad). The amplified transcript level of each specific gene was normalized on CHMP2A housekeeping. ΔΔCt quantification method was used for RT-qPCR analyses. The list of primers used is provided in Supplementary Table 2.

### Western blot

Western blot analysis was performed using standard techniques^8^.

The primary antibodies were: HELLS (Rabbit mAb#7998, 1:1000 Cell Signaling Technology), γ PAK2 (E-9 Mouse mAb sc-373740, 1:1000 Santa Cruz Biotechnology, Inc), RHOA (26C4 Mouse mAb sc-418, 1:1000 Santa Cruz Biotechnology, Inc), RHOU (Rabbit, PA5-69128, 1:500 Invitrogen), YY1 (Rabbit, D3D4Q, 1:1000, Cell Signaling Technology), β-tubulin antibody (sc-23949, 1:100, Santa Cruz Biotechnology, Inc) and GAPDH (Rabbit mAb#2118, 1:2000, Cell Signaling Technology)

All secondary antibodies (rabbit and mouse) were HRP-conjugated (GE Healthcare) and diluted 1:3000.

Densitometric analysis was performed using the ImageJ software.

### Immunofluorescence (IF)

Cells were spotted on glass slides using Cytospin (Thermo Scientific), fixed with 4% paraformaldehyde for 10 min and permeabilized with 0.1% Triton X-100 for 3 min. Dots were blocked in 1% PBS-BSA solution for 40 min at room temperature and incubated with phalloidin (Alexa Fluor^®^ 488, Thermo Fisher) for actin staining for 50 min. Dots were washed in PBS for three times and nuclei were stained with DAPI. For microtubules staining we used β-tubulin antibody (sc-23949, 1:100, Santa Cruz Biotechnology, Inc). Immunofluorescences were detected with Nikon Eclipse (Ni) microscope using 60X.

### Chromatin immunoprecipitation (ChIP)

ChIP was performed as previously described^8^. Chromatin was precipitated with antibodies against HELLS (4ug, Rabbit Polyclonal, orb178580, Biorbyt), YY1 (D3D4Q, 1:100, Cell Signaling Technolgy), or IgG-isotype control (#66362, Cell Signaling Technology). Each qRT-PCR value was normalized over the appropriate input control and reported in graphs as a relative fold on IgG.

The list of primers used is provided in Supplementary Table 2.

### Co-Immunoprecipitation (Co-IP)

Cells were collected, crosslinked with 1% formaldehyde for 10 min, treated with 1.25 M glycine for 5 min and resuspended in Buffer A (10 mM HEPES pH 7.9, 1.5 mM MgCl2, 10 mM KCl, 0.5% NP-40) supplemented with protease inhibitor for 8 min in ice. After centrifugation at 3000 rpm for 2 min, the supernatant was collected as the cytoplasmic fraction and used as the quality control of the experiment. The pellet was washed two times with BUFFER B (10 mM HEPES pH7.9, 1.5 mM MgCl2, 10 mM KCl), centrifuged at 3000 rpm for 2 min. Nuclei were resuspended in lysis buffer (50 mM Tris-HCl pH 7.4, 150 mM NaCl, 1 mM EDTA, 1% Triton X-100) supplemented with protease inhibitor and kept for 1 h in rotation at 4°C. Nuclei extracts were sonicated using a Bioruptor^®^ Pico sonicator (Diagenode) and centrifuged at 16,000 *g* for 10 min. Supernatant was kept and was quantified with Bradford. For each experiment, 4 mg of nuclei extracts was used for immunoprecipitation and 150ɥg was kept as input control. Precoating step was performed using Protein A-Sepharose^®^ CL-4B beads (GE Healthcare, Sigma Aldrich), HELLS antibody (Rabbit Polyclonal, orb178580, Biorbyt). Preclearing step was performed using total nuclear lysate and Protein A-Sepharose beads for aspecific removal for 1 h in rotation at 4°C. After centrifugation at 500 *g* for 5 min, we combined the beads from precoating and supernatant from preclearing steps and kept in rotation overnight at 4°C. After centrifugation of 500 *g* for 5 min, the supernatant was discarded and washed four times with TBS 1X (50 mM Tris-HCl pH 7.4, 150 mM NaCl). Laemmli Sample Buffer 4x (Biorad) was added to the immunoprecipitated and samples were boiled for 20 min. Co-IP was detected by western blot using the secondary antibody mouse anti-rabbit IgG HRP conjugate (L27A9) (#5127, 1:2000, Cell Signaling Technology).

### Library preparation and RNA-sequencing

RNA seq libraries were obtained starting from 500 ng of total RNA following Illumina TruSeq Stranded TotalRNA preparation protocol. Sequencing was performed using Illumina NEXSeq high-output cartridge (double-stranded, reads length 75bp-2 ×75).

A sequencing depth of at least 60 million reads for each sample was guaranteed.

Sequencing quality was assessed using the FastQC v0.11.8 software (www.bioinformatics.babraham.ac.uk/projects/fastqc/), showing on average a Phred score per base >34 in each sample. Raw sequences were then aligned to the human reference transcriptome (GRCh38, Gencode release 30) using STAR version 2.7^23^ and gene abundances were estimated with RSEM algorithm (v1.3.1)^24^. Differential expression analysis was performed using DESeq2 R package^25^, considering a False Discovery Rate (FDR) of 10% and excluding genes with low read counts. Heatmap representation and unsupervised hierarchical clustering with a complete linkage method were exploited to graphically depict differentially expressed genes (FRD < 0.1).

Significant genes underwent enrichment analysis, performed on Gene Ontology biological processes, KEGG and Reactome pathways databases via enrichR package^26^, using a significance threshold of 0.05 on *p*-value adjusted by Benjamini–Hochberg correction for multiple testing.

### Transcriptional factors motif enrichment

For transcriptional factor motif search, JASPAR 2020 and PROMO (version 3.0.2) software tools were used. A motif similarity threshold of 80% and a dissimilarity level of 15% were respectively applied for JASPAR 2020 and PROMO prediction results.

### Statistical analysis

Statistical analyses were performed using the GraphPad Prism Software (GraphPad). Statistical significance was determined using Student’s *t* test. Each experiment was replicated multiple time (>3 up to 6).

## Results

### HELLS controls ALK^−^ALCL proliferation by transcriptionally coordinating a panel of cytoskeleton related genes involved in cytokinesis

**Fig. 1:**
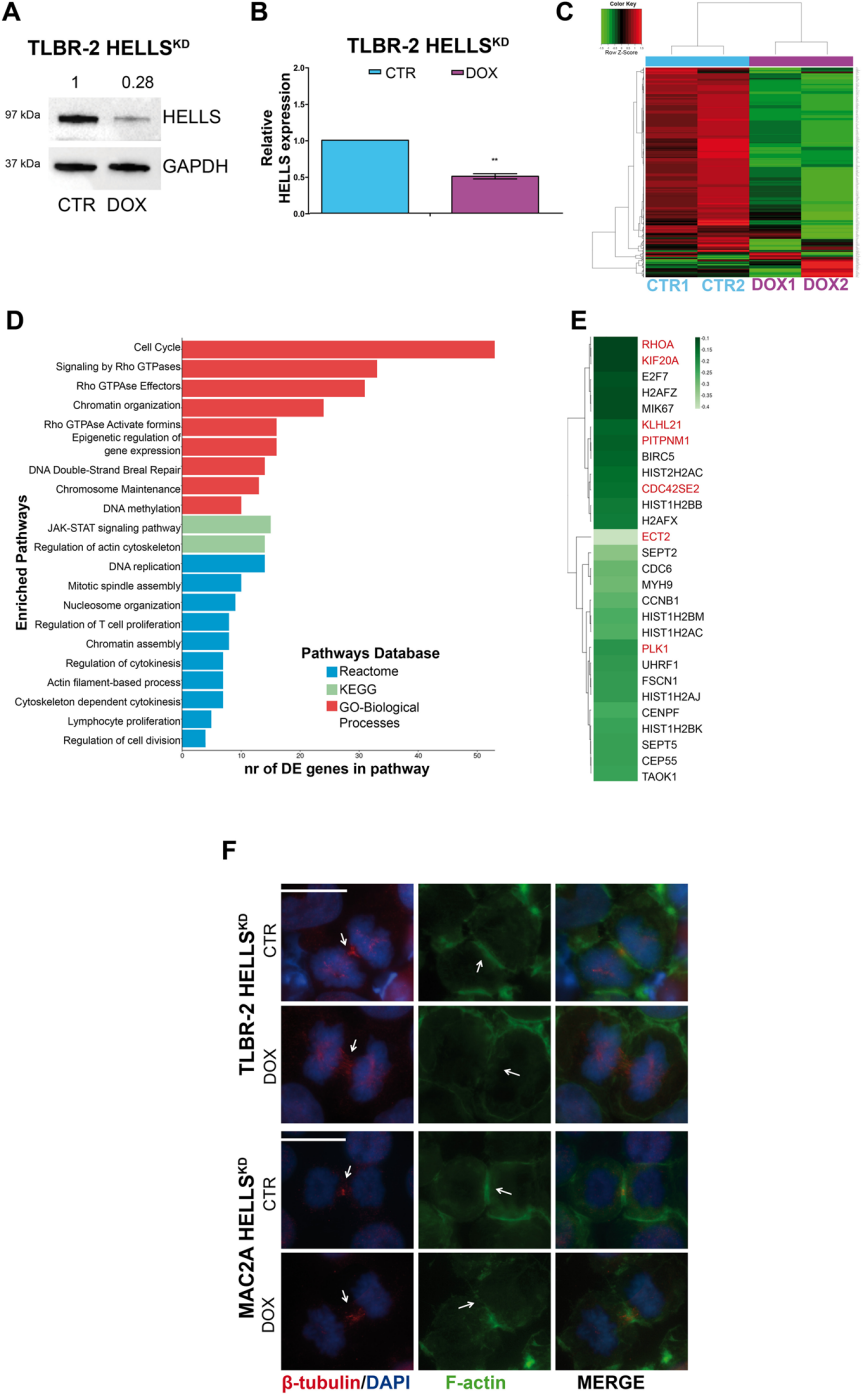
HELLS transcriptionally controls cytokinesis. **A** Western blot shows HELLS expression in TLBR-2 HELLS^KD^ cells after 48 h of doxycycline (DOX) induction. GAPDH was used as housekeeping gene. **B** qRT-PCR analysis of HELLS expression in TLBR-2 HELLS^KD^ cells after 48 h of doxycycline induction. The values represent mean ± SEM (*n* = 3) *p < 0.05; **p < 0.01. **C** The heatmap depicts hierarchical clustering based on the 728 differentially expressed genes, whose read counts are Z-score normalized. Unsupervised hierarchical clustering was performed between DOX and CTRL samples (as indicated by the colored bar on columns) with a complete linkage method. Color intensity for each gene shows Z-score values ranging from red for upregulation and green for downregulation **D** Most significant enriched pathways (adjusted p-value<0.05) are represented showing the number of DE genes mapped in each considered pathway. **E** The heatmap depicts validated significantly downregulated genes. Green color bar shows fold difference on Log_2_ scale calculated between DOX and CTRL samples. Darker green represents the most downregulated genes. Genes in red were selected for further validations. **F** Immunofluorescence images of TLBR-2 HELLS^KD^ cells and MAC2A HELLS^KD^ cells after 48 h of doxycycline (DOX) induction. Cells were stained with DAPI, F-actin, and β-tubulin antibodies. The white scale bar represents 10 μm.

To get insight into the transcriptional regulation of HELLS, we performed an RNA-sequencing profiling in TLBR-2 cells which represent the ALK^−^ALCL subtype known as Breast Implanted Associated (BIA)-ALCL^8,27^. We generated inducible HELLS knockdown (KD) lines (TLBR-2 HELLS^KD^) using doxycycline (DOX)-inducible shRNA. HELLS KD was assessed by WB and qRT-PCR (Fig. 1A, B) and functionally validated by the reduction in the expression of already described HELLS-downstream targets^28^ (Supplementary Fig. 1A). After DOX induction, the gene expression profile of TLBR-2 HELLS^KD^ cells was analyzed and compared to the one obtained from untreated cells used as control. Transcriptional changes observed after HELLS^KD^ revealed 728 differentially expressed genes. 413 were downregulated and 315 were upregulated upon HELLS^KD^ with a FDR < 0.1 (Fig. 1C). Gene ontology analysis of HELLS-target genes revealed the enrichment of several categories including cell cycle, DNA damage, histone modification, and chromatin organization. Noticeably, top scoring in this list, there were multiple Rho GTPases and cytoskeleton related categories, including cytoskeleton regulation by Rho GTPases and Rho GTPases signaling (Fig. 1D, E). This was particularly interesting since we previously reported that the reduced cellular proliferation displayed by ALK^−^ALCL upon HELLS loss is associated with defects in cytokinesis and a marked increase in multi-nucleated cells of which Rho-GTPases are major players^8^.

To give a phenotypic readout to these results, we evaluated the organization of cytoskeleton in T-cells^29^ performing immunostaining for β-tubulin (as a principal constituent of microtubules) and for F-actin upon HELLS^KD^ in TLBR-2 and in an additional cell line representing the systemic ALK^−^ALCL subtype (MAC2A) (Supplementary Fig. 1B, C). Although we did not observe differences in the quantity of β-tubulin (Supplementary Fig. 1D), a less organized localization of β-tubulin in the midzone of central spindle was detected in HELLS^KD^ cells as compared to control cells (Fig. 1F). By contrast, we observed a profound reorganization of the F-actin with a reduced alignment and compaction in cleavage furrow structure. This phenomenon resulted in an incomplete actomyosin ring formation in HELLS^KD^ cells as compared to control cells (Fig. 1F), in agreement with the multi-nucleated phenotype previously reported^8^.

Together, these results indicate that, in ALK^−^ALCL, HELLS contributes to an appropriate cytokinesis via the transcriptional control of genes involved in contractile ring regulation.

### HELLS controls cytokinesis by directly binding to target gene promoters

**Fig. 2:**
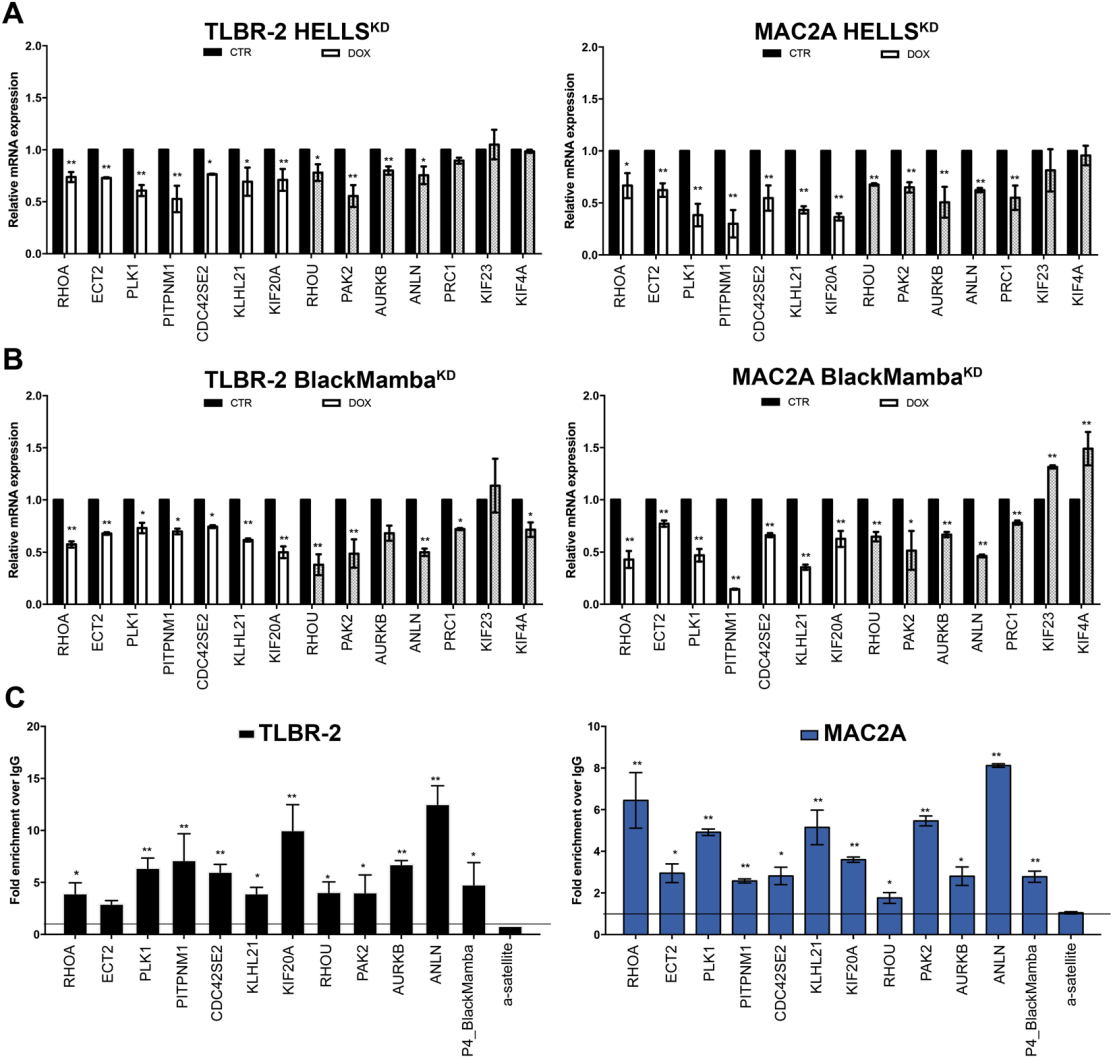
HELLS binds target gene promoters. **A** qRT-PCR validation of significantly downregulated genes obtained from RNA-sequencing. White bars represent the most significantly downregulated genes (FDR < 0.1) and gray bars represent less significant downregulated genes (FDR > 0.1) in TLBR-2 HELLS^KD^ cells and MAC2A HELLS^KD^ cells after 48 h of doxycycline (DOX) induction. Each data represent mean ± SEM (*n* = 3). Two-tailed *t*-test. *p < 0.05; **p < 0.01. **B** qRT-PCR validation of significantly downregulated genes obtained from RNA-sequencing. White bars represent the most significantly downregulated genes (FDR < 0.1) and gray bars represent less significant downregulated genes (FDR > 0.1) in TLBR-2 BlackMamba^KD^ cells and MAC2A BlackMamba^KD^ cells after 6 days of doxycycline (DOX) induction. Each data represent mean ± SEM (*n* = 3). Two-tailed *t*-test. *p < 0.05; **p < 0.01. **C** ChIP qRT-PCR detection of HELLS antibody in a panel of target gene promoters in TLBR-2 and MAC2A.P4_BlackMamba and α-satellite were used as positive and negative controls, respectively. The values represent the relative fold enrichment over IgG and are indicated as mean ± SEM (*n* = 3). Two-tailed *t*-test. *p < 0.05; **p < 0.01.

Using RT-qPCR, we validated a representative set of altered genes involved in cytoskeleton and cytokinesis confirming the RNA-sequencing results and the effect of HELLS KD on these processes (Fig. 2A, Supplementary Fig. 2A). Since we showed that HELLS cooperates with the lncRNA BlackMamba for transcriptional activity in ALK^−^ALCL and concurs to the lncRNA BlackMamba pro-oncogenic role in these cells^8^, we investigated whether lncRNA BlackMamba was involved in the HELLS-dependent regulation of these genes. Taking advance of the previously generated TLBR-2 and MAC2A BlackMamba^KD^ inducible cell lines^8^, we showed that silencing of this lncRNA resulted in an important reduction of all tested HELLS-target genes (Fig. 2B, Supplementary Fig. 2B, C) confirming the functional synergy between BlackMamba and HELLS and further indicating the cytoskeleton related genes as a central node of the transcriptional program supported by this axis in ALK^−^ALCL.

To further investigate the direct role of HELLS in the regulation of these genes, we performed ChIP experiments to assess the binding of HELLS on their promoters. We observed a significant and specific enrichment of HELLS binding on 10 out of 11 promoter regions tested in both MAC2A and TLBR-2 cells (Fig. 2C). These data confirm that HELLS acts as a transcriptional activator of these genes.

### YY1 is a transcriptional partner of HELLS in ALK^−^ALCL

**Fig. 3:**
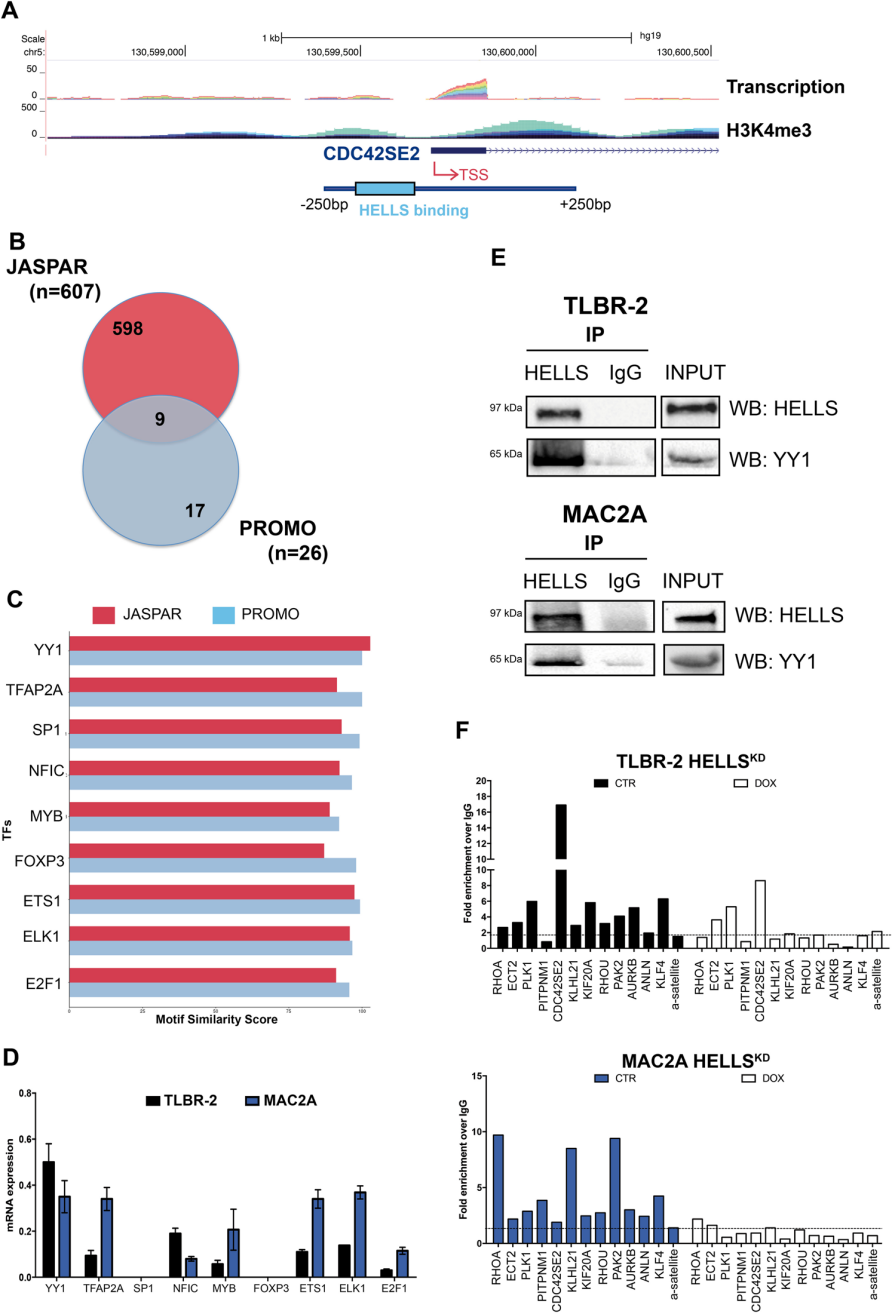
HELLS interacts with the transcriptional factor YY1. **A** Schematic representation of CDC42SE2 genomic locus showing the position, the level of transcription and the enrichment of H3K4me3 on its putative promoter. **B** The Venn diagram represents the intersection between transcriptional factors (TFs) identified by JASPAR (*n* = 607) and PROMO (*n* = 26) TFs binding prediction tools. The intersection shows the number of HELLS-predicted and common TFs (*n* = 9). **C** JASPAR and PROMO similarity scores are represented for top-ranked enriched transcription factors (TFs). **D** qRT-PCR analysis of top scoring TFs in TLBR-2 and MAC2A cell lines. Each data represent mean ± SEM (*n* = 3). **E** Nuclear extract from TLBR-2 and MAC2A cells were tested for the presence of a multi-protein complex. Co-immunoprecipitation experiments were performed to evaluate binding of HELLS to YY1. Western blots are representative of two independent experiments. **F** ChIP qRT-PCR detection of YY1 in TLBR-2 HELLS^KD^ and MAC2A HELLS^KD^ after 48 h of doxycycline (DOX) induction. KLF4 and α-satellite were used as positive and negative controls, respectively. The values represent the fold enrichment over IgG (representative of three independent experiments).

Little is still known on the transcriptional function of helicases. Thus, to explore how HELLS controls the expression of its target genes we searched for putative transcriptional factors (TFs) able to cooperate with HELLS in ALK^−^ALCL. A region spanning 500 bp around the transcriptional starting site (TSS) of each HELLS-target gene was selected (Fig. 3A). A prediction search for TF binding sites enriched in these regions was performed using JASPAR^30^ and PROMO^31^ tools. 607 TFs and 26 TFs were significantly identified by JASPAR and PROMO, respectively. Merge of these lists resulted in a final list of 9 top scoring TFs and HELLS potential co-factors (predicted to bind up to 90% of the promoters regions used in this search): Yin Yang 1(YY1), Transcription Factor AP-2 Alpha (TFAP2A), Sp1 transcriptional factor (SP1), Nuclear Factor 1C (NFIC), MYB Proto-Oncogene Transcription Factor (MYB), Forkhead Box P3 (FOXP3), ETS Proto-Oncogene 1 (ETS1), ETS Transcription Factor ELK1 (ELK1) and E2F Transcription Factor 1(E2F1) (Fig. 3B, C).

To validate this analysis, we first tested the basal expression of each of these TFs in ALK^−^ALCL cell lines. 7 out of 9 (78%) TFs were expressed in all tested cell lines even if with variable levels. By contrast, no expression was observed for FOXP3 and SP1 (Fig. 3D).

Next, we investigated the potential interaction of these factors with HELLS by co-immunoprecipitation experiments in nuclear extracts. Among all the TFs investigated, only YY1 was found to interact with HELLS in both TLBR-2 and MAC2A cells (Fig. 3E), although the levels of YY1 in MAC2A cells are significantly lower than in TLBR2. To enforce the idea of cooperation between HELLS and YY1, we performed ChIP assay to investigate the binding of YY1 on the promoter regions of HELLS-target genes both in MAC2A and TLBR-2 cell lines. We showed that 80% of the tested promoter (9/11) were simultaneously bound by both factors supporting the hypothesis of a functional cooperation between these two factors (Fig. 3F). To establish a hierarchy within their relationship, we analyzed how HELLS KD affects YY1 binding on target promoters. Noticeably, YY1 was displaced by these regions in the absence of HELLS while no perturbation of YY1 expression levels was detected upon HELLS KD (Fig. 3F, Supplementary Fig. 3A) indicating that HELLS is crucial in positioning YY1 on specific target genes in the context of ALK^−^ALCL.

### YY1 cooperates with HELLS to foster the transcription of cytokinesis-related genes

**Fig. 4:**
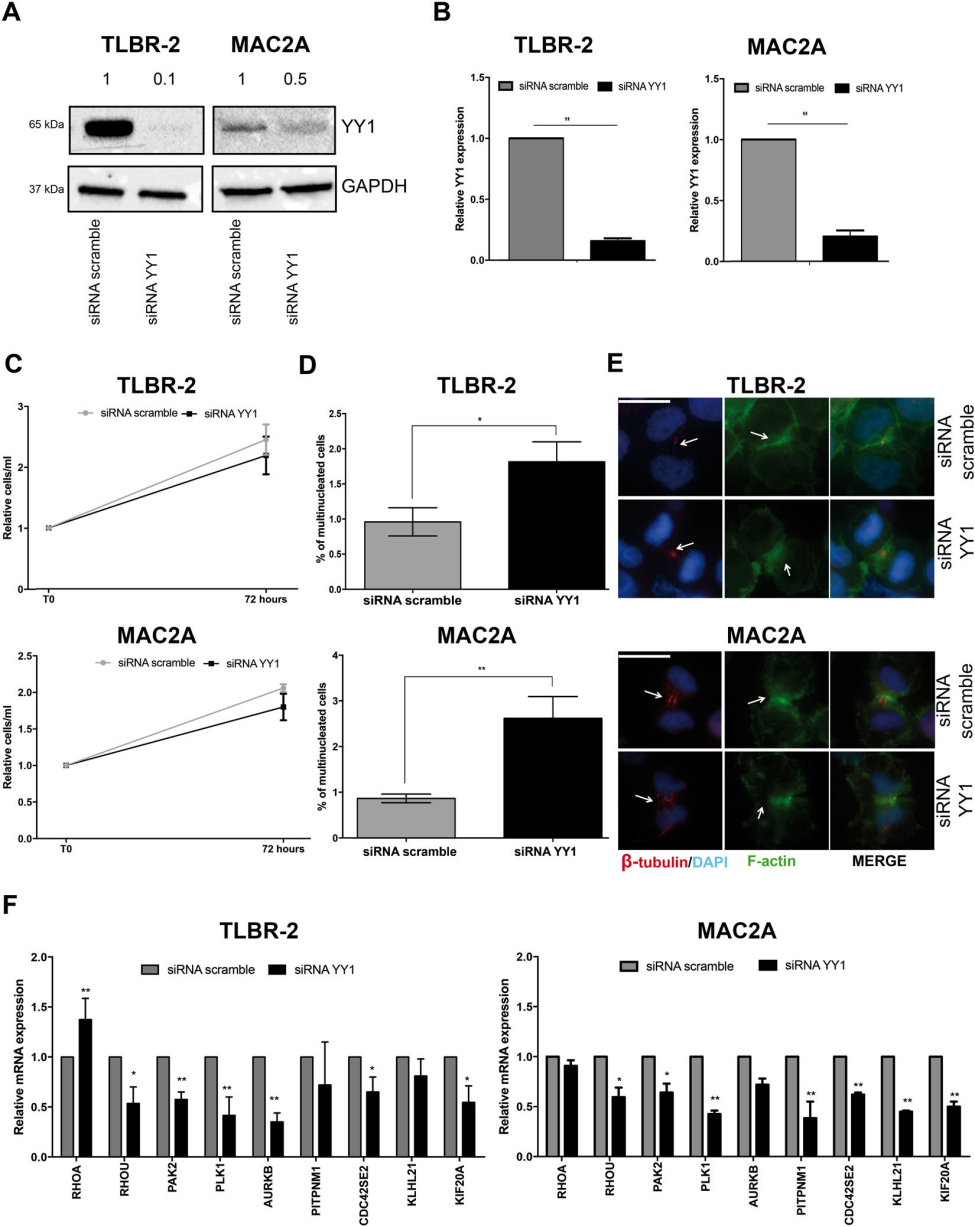
YY1 cooperates with HELLS to regulate the multi-nucleated phenotype. **A** Western Blot shows the knockdown of YY1 36 h post-nucleofection with a specific siRNA in TLBR-2 and MAC2A. GAPDH was used as housekeeping gene. **B** qRT-PCR analysis of YY1 expression after siRNA in TLBR-2 and MAC2A cell lines (36 h post-nucleofection). Each data represent mean ± SEM (*n* = 3). Two-tailed *t*-test. **p < 0.01. **C** The graph represents the relative growth curve of TLBR-2 and MAC2A 72 h post-nucleofection with YY1 specific siRNA. Data were normalized on siRNA scramble values. Each data point represents the mean ± SEM (*n* = 3). Two-tailed *t*-test**. D**. The panels show the percentage of multi-nucleated cells in at least 500 cells stained with β-tubulin and F-actin antibodies (48 h post-nucleofection). Each data point represents the mean ± SEM (*n* = 3). Two-tailed *t*-test. *p < 0.05; **p < 0.01. **E** Panels show representative immunofluorescences of TLBR-2 and MAC2A stained with DAPI, F-actin, and β-tubulin antibodies 48 h post-nucleofection. The scale bar represents 10 μm. **F** qRT-PCR analysis of a panel of selected HELLS-target genes in TLBR-2 and MAC2A 36 h post-nucleofection with specific YY1 siRNA. The values represent mean ± SEM (*n* = 3). Two-tailed *t*-test. *p < 0.05; **p < 0.01.

YY1 is an ubiquitous transcriptional factor known to have a fundamental role in normal and cancer-related processes^32^. Thus, to consolidate its role as HELLS partner in the transcriptional program of ALK^−^ALCL, we silenced YY1 with specific siRNA. WB analyses and qRT-PCR confirmed the efficiency of the silencing (Fig. 4A, B). Parallel analysis demonstrated that previously reported YY1 target genes^33–35^ were coherently altered upon its KD (Supplementary Fig. 3B, C). Proliferation analysis of TLBR2 and MAC2A transfected with either siRNA against YY1 or scramble oligos as control did not evidence a significant effect on cell proliferation (Fig. 4C). However, we observed a significant increase in the number of multi-nucleated cells in YY1^KD^ (Fig. 4D, E).

Coherently, silencing of YY1 was associated with repression of RHOU, PAK2, PLK1, AURKB, PITPNM1, CDC42SE2, KLHL21, and KIF20A (Fig. 4F**)**. This was in line with a positive cooperation of YY1 and HELLS in the regulation of cytokinesis genes and with the observed increase in multi-nuclei. Intriguingly, YY1 did not affect RHOA expression suggesting that other factors participate to HELLS transcriptional regulation of ALK^−^ALCLs. The involvement of other transcriptional partners likely accounts for the fact that YY1^KD^ only partially recapitulates HELLS^KD^ phenotype.

### Downstream effects of HELLS are mediated by multiple Rho-GTPases and effectors

Rho-GTPases are a family of small GTPase proteins involved in many aspects of intracellular actin dynamics including cell division. During the cytokinesis, Rho-GTPases trigger the initiation of the cleavage furrow from which cytosol division begins^36–39^.

**Fig. 5:**
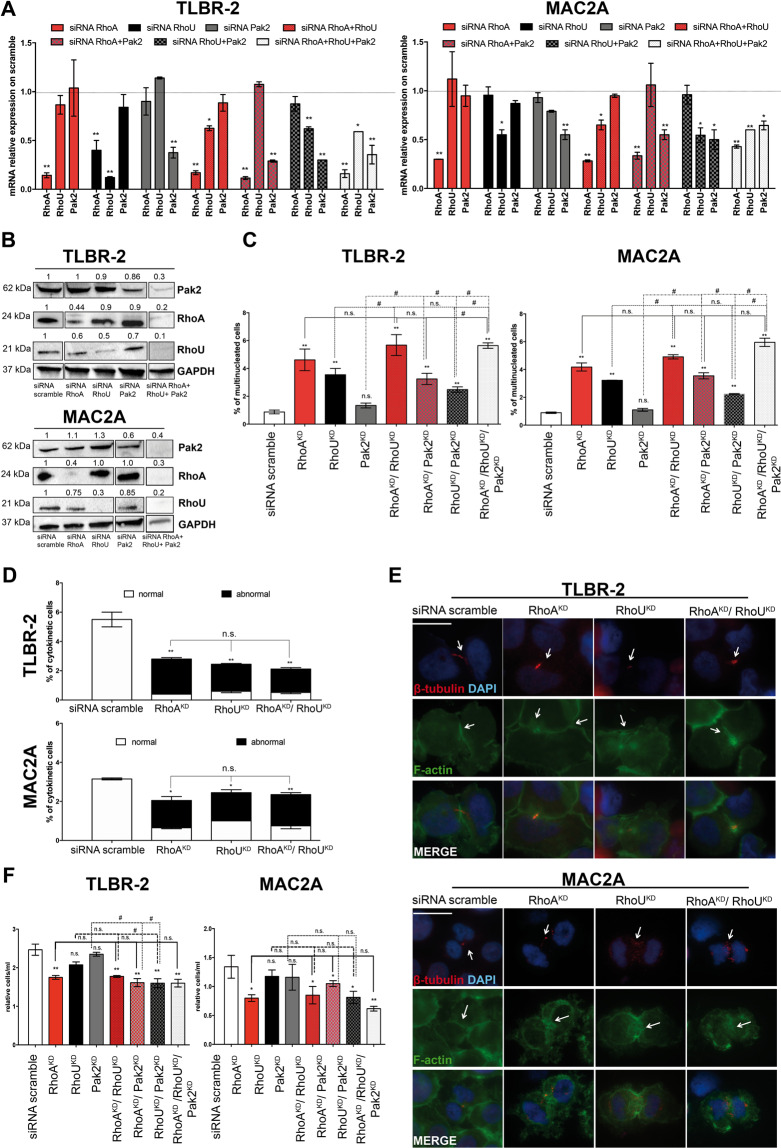
Rho-GTPases mediate HELLS-downstream effects. **A** qRT-PCR analysis of RhoA, RhoU and Pak2 expression after single or combined siRNAs in TLBR-2 and MAC2A cell lines (36 h post-nucleofection). Each data represent mean ± SEM (*n* = 3). Two-tailed *t*-test. *p < 0.05, **p < 0.01. **B** Western blots show knockdown of RhoA, RhoU, or Pak2 36 h post-nucleofection with specific siRNAs in TLBR-2 and MAC2A. GAPDH was used as housekeeping gene. **C** The histograms show the percentage of multi-nucleated cells in at least 500 cells TLBR-2 and MAC2A stained with DAPI, F-actin, and β-tubulin antibodies (48 h post-nucleofection). Cells were nucleofected with single and combined specific siRNAs against RhoA, RhoU, and Pak2. Each data point represents the mean ± SEM (*n* = 3). Two-tailed *t*-test. *p < 0.05 and **p < 0.01 relative to siRNA scramble. # *p* < 0.05 relative to RhoA^KD^, RhoU^KD,^ or Pak2^KD^. **D** The histograms show the percentage of cytokinetic cells in at least 500 cells TLBR-2 and MAC2A stained with DAPI, F-actin, and β-tubulin antibodies (48 h post-nucleofection). The term “abnormal” refers to cytokinetic cells with defects in cleavage furrow and/or microtubules structures. Cells were nucleofected with single and combined specific siRNAs against RhoA and RhoU. Each data point represents the mean ± SEM (*n* = 3). Two-tailed *t*-test. *p < 0.05 and **p < 0.01 relative to siRNA scramble. **E** Immunofluorescence of TLBR-2 and MAC2A nucleofected with siRNA scramble and single and combined RhoA and RhoU siRNAs (48 h post-nucleofection). Cells were stained with DAPI, F-actin, and β-tubulin antibodies. The scale bar represents 10 μm. **F** Relative growth curve of TLBR-2 and MAC2A nucleofected with siRNA scramble and single or combined RhoA and RhoU and Pak2 siRNAs (72 h post-nucleofection). Each data were normalized to siRNA scramble and represent the mean ± SEM (*n* = 3). Two-tailed *t*-test. *p < 0.05 and **p < 0.01 relative to siiRNA scramble. # *p* < 0.05 relative to RhoA^KD^, RhoU^KD^, or Pak2^KD^.

Rho-GTPases are also known for their role in the regulation of cell growth and proliferation and cytoskeleton rearrangements in T-lymphomas^40^. Thus, to consolidate the relevance of the transcriptional program associated with HELLS, we investigated the relevance of these genes in ALK^−^ALCL. To this end, we knocked down the expression of RhoA, RhoU, and Pak2 with specific siRNAs. Since Rho-GTPases are known to work in a highly cooperative manner^41–43^, we also combined siRNAs to simultaneously knockdown two or more of these proteins. Figures 5A and 5B show the efficiency of the silencing at the mRNA and protein level in both MAC2A and TLBR-2 cells.

We noticed that while RhoA^KD^ or Pak2^KD^ did not alter the expression of the others, RhoU ^KD^ exerted a significant effect on RhoA expression, suggesting a tight interplay between these two proteins.

Morphologically, RhoA^KD^ and RhoU^KD^ led to the increase in the percentage of multi-nucleated cells relative to siRNA scramble. By contrast, no such effect was observed for Pak2 silencing. This phenomenon slightly increased when RhoA^KD^ and RhoU^KD^ were combined. On the contrary, combined RhoU^KD^/Pak2^KD^ and RhoA^KD^/Pak2^KD^ did not increase the percentage of multi-nucleated cells relative to RhoU^KD^ and RhoA^KD^, respectively. Consistently, triple KD resulted in an induction of multinuclei comparable to combined RhoA^KD^/RhoU^KD^ or RhoA^KD^ (Fig. 5C). To assess if the multi-nucleated phenotype resulted from cytokinesis failure^44^, we quantified cytokinetic cells by immunofluorescence using F-actin and β-tubulin staining. A significant decrease in the percentage of total cytokinetic cells and a relative increase in abnormal cytokinetic cells were observed in single and combined RhoA^KD^/RhoU^KD^ compared to siRNA scramble (Fig. 5D).

Immunofluorescences showed that both RhoA^KD^ and RhoU^KD^ affected β-tubulin organization at central spindles or midbody structures. The proper F-actin organization at contractile ring was also dramatically affected resulting in an abnormal formation of cleavage furrow (Fig. 5E). As expected, combined RhoA^KD^ and RhoU^KD^ resulted in a more pronounced phenotype close to the one obtained with HELLS^KD^ (Fig. 5E).

The analysis of cell proliferation was coherent with these observations. Single silencing of RhoU and Pak2 did not affect significantly cell growth while a significant decrease in cell proliferation was observed in RhoA^KD^ in both cell models. Combined RhoU^KD^/RhoA^KD^ resulted in a cell proliferation reduction similarly to single RhoA^KD^ whereas combined RhoU^KD^/Pak2^KD^ enhanced the effects of single RhoU^KD^ and Pak2^KD^ but only in TLBR-2. Notably, triple KD resulted in a significant decrease in cell proliferation, but this reduction was similar to RhoA^KD^ or RhoU^KD^/Pak2^KD^ (Fig. 5F). Collectively, these data demonstrated that RhoA and RhoU mediate HELLS effects on cell proliferation and cell division of ALK^−^ALCLs and that RhoA has a prominent role in this process.

## Discussion

Aberrant expression of epigenetic modifiers fostering the transcriptional program of neoplastic T-cells is emerging as a common feature and potential vulnerability of ALK^−^ ALCLs^45,46^.

**Fig. 6:**
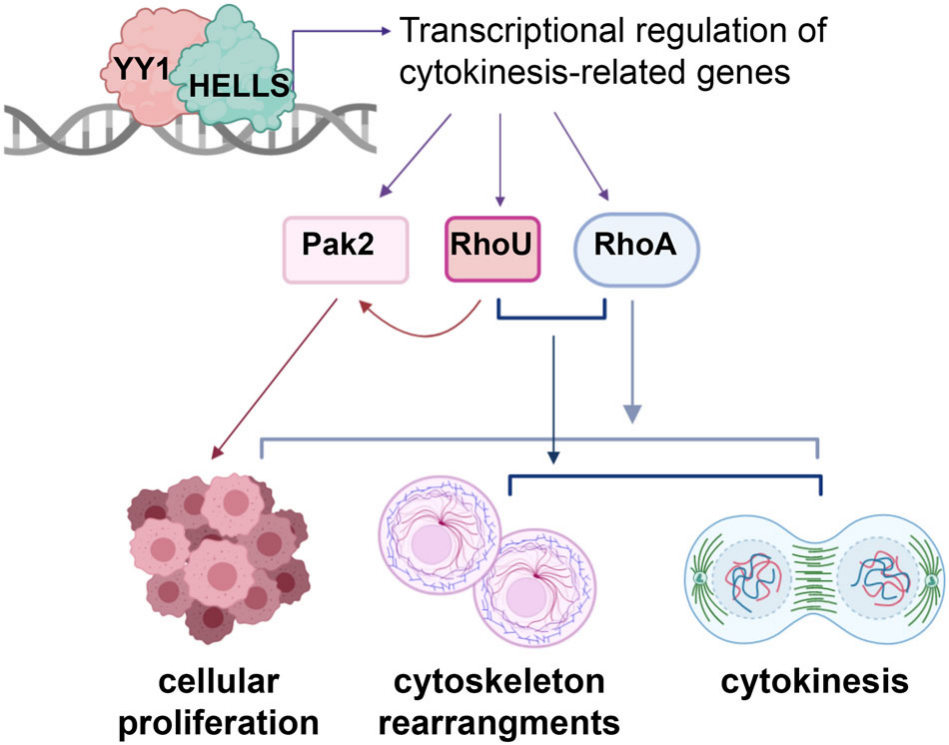
Proposed molecular mechanism. Schematic representation of HELLS role in the regulation of ALK^−^ALCLs cytokinesis-related program (created with BioRender.com).

In line with this evidence, here we showed that the DNA-helicase HELLS supports ALK^−^ALCL proliferation by controlling a gene expression program that is functional for the execution of cytokinesis and therefore for proficient cell division (Fig. 6).

HELLS is a multifunctional protein proved to play, among the others, critical roles in DNA methylation, chromatin packaging, and development of lymphoid tissue^47^. Known also as Lymphoid-specific helicase (Lsh) HELLS is required for normal development and survival of lymphoid and other tissues via chromatin organization^48^^49^, promotion of DNA double-strand break repair^50,51^ and chromatin accessibility modification^52,53^. In cancer, HELLS is deregulated in several settings i.e. gliomas^54^, retinoblastoma^55,56^, prostate^28^, breast carcinomas^57,58^, medulloblastoma^59^, leukemia^60^ where it promotes cellular proliferation and stemness. A significant part of HELLS activity in these processes is mediated by its transcriptional function. The way through which HELLS controls gene expression is still partially undefined. Its interaction with epigenetic silencers including G9a^61^ and DNMTs^62^ as well as with transcriptional factors like E2F3^28,56^ and c-Myc^54^ has been described. Here, we added an additional part of information showing that in the specific context of ALK^−^ALCL, HELLS interacts and functionally cooperates with the transcription factor YY1. We demonstrate that DNA binding motif for YY1 is enriched within the HELLS binding sites and that these two factors physically interact and co-occupy the same regions at the level of target promoters to ensure transcription of a large set of cytokinesis-related genes.

YY1 is an ubiquitously expressed transcriptional factor with a fundamental role in embryogenesis, adult hematopoiesis, differentiation, replication, and cellular proliferation^32,63,64^. YY1 ensures the proper completion of mitosis and its knockdown leads to defects in cytokinesis and accumulation of multi-nucleated cells^65^. YY1 is often deregulated in hematopoietic malignancies where it controls the survival and growth of neoplastic cells^66,67^.

Coherently, here we reported that in ALK^−^ALCL loss of YY1 is associated with accumulation of multi-nucleated cells likely attributable to incomplete cytokinesis.

The functional relationship between HELLS and YY1 has never been described before. Interestingly, we showed that the loss of HELLS leads to YY1 displacement from DNA. This seems to indicate that HELLS, by altering chromatin accessibility, works by priming the binding of specific transcription factors to target promoters therefore regulating specific set of context specific genes.

By RNA-sequencing profiling we showed that a significant part of the HELLS transcriptional program in ALK^−^ALCL converges on the regulation of cytoskeleton and cytokinesis. To the best of our knowledge this is the first time that the transcriptional activity of HELLS is linked to these biological processes.

Cytoskeleton is the structure responsible for cell shape maintenance and organization. It also confers mechanical support to every cellular process from proliferation and division to cell migration, adhesion, and interaction with the surrounding microenvironment.

Of note, recent genetic and molecular profiling studies have unveiled a critical role of cytoskeleton during transformation and progression of T-cells^68,69^. Although we still lack of a definitive overall view of how cytoskeleton change during lymphomagenesis, the emerging picture suggests that the cytoskeleton transcends the maintenance of cell morphology and polarity providing a more complex support to T-cells in the response to intrinsic and environmental clues.

Among HELLS-downstream effectors, we identified several Rho-GTPases and their related proteins including RhoA, RhoU, and Pak2. Single and combined KDs of these proteins highlight that RhoA and RhoU are key effectors of HELLS program controlling both cell proliferation and cell division.

RhoA is a key player in T-cell processes^70^ and its deregulation has emerged as a central issue of T-lymphoma biology^68,70–73^. Coherently, our results demonstrate that RhoA is a fundamental effector of HELLS-dependent oncogenic program. At the transcriptional level, RhoA results regulated by HELLS but not by YY1 suggesting a more complex regulation of this Rho-GTPase in this setting. Limited information are available on the regulation of RhoA^74^, and given its centrality, additionally studies are needed to better clarify this point.

As RhoU can mediate the effects of WNT and STAT3 signaling pathways in regulating cell morphology, cytoskeletal organization, and proliferation^75,76^, our data provide a new layer of complexity demonstrating a new role of RhoU in cytokinesis via the STAT3-BlackMamba-HELLS axis.

The cooperative role of Rho-GTPases in the execution of neoplastic program driven by HELLS is in agreement with their fundamental role in the regulation of cell proliferation, cell division, and actin polymerization in cancer^77^.

Collectively, our data provide novel insights into the mechanism sustaining the progression of ALK^−^ALCL via the untapped role of HELLS as transcriptional regulator of cytokinesis. Since HELLS is expressed in many tumors and plays a relevant role in the transcription and genomic stability of cancers, its pharmacological inhibition may represent a promising therapeutic strategy in lymphomas and in other human neoplasms.

## Supplementary information

Supplemental file

Supplemental figure 1

Supplemental figure 2

Supplemental figure 3

## Data Availability

Raw data files of RNA-sequencing have been deposited in EMBL-EBI ArrayExpress and are accessible through the accession number E-MTAB-9918.
