# Supplementary material for: The DNA-helicase HELLS drives ALK− ALCL proliferation by the transcriptional control of a cytokinesis-related program
Source: Cell Death Dis. 2021 Jan 27;12(1):130. doi: 10.1038/s41419-021-03425-0 (PMC7840974; doi:10.1038/s41419-021-03425-0)
Supplement: Supplementary file 1 — Supplemental file [file 41419_2021_3425_MOESM1_ESM.doc]

**Supplementary Figure Legends**

**Figure S1**. HELLS KD analysis.

A. qRT-PCR analysis of E2F1 and MLL1 (used as positive controls of HELLS knockdown) in TLBR-2 HELLS^KD^ after 48 hours of doxycycline (DOX) treatment. The values represent mean ± SEM (n = 3). Two-tailed t-test. **p <0.01.

B. Western blot analysis of HELLS expression in MAC2A HELLS^KD^ after 48 hours of doxycycline induction.

C. qRT-PCR analysis of HELLS and its targets E2F1 and MLL1 in MAC2A HELLS^KD^ after 48 hours of doxycycline (DOX) treatment.

D. Western blot analysis of β-tubulin in TLBR-2 HELLS^KD^ and MAC2A HELLS^KD^ after 48 hours of doxycycline induction.

**Figure S2**. BlackMamba KD analysis.

A. qRT-PCR analysis of a panel of target genes in TLBR-2 plain after 48 hours of doxycycline treatment (DOX).

B-C. qRT-PCR analysis of BlackMamba and HELLS expression, used as positive control, in TLBR-2 HELLS^KD^ and MAC2A HELLS^KD^ after 6 days of doxycycline treatment. The values represent mean ± SEM (n = 3). Two-tailed t-test. **p <0.01.

**Figure S3**. YY1 analysis.

A. qRT-PCR analysis of YY1 expression in TLBR-2 HELLS^KD^ and MAC2A HELLS^KD^ after 48 hours of doxycycline induction (DOX). The values represent mean ± SEM (n = 3). Two-tailed t-test. n.s.= not significant.

B-C. qRT-PCR analysis of YY1 targets IL-6, KLF4 and MYC in TLBR-2 and MAC2A nucleofected with specific YY1 siRNA (36 hours post nucleofection).

**Supplementary Table Legends**

**Supplemental Table 1**

List of shRNAs sequences used in this paper

**Supplemental Table 2**

List of primers used for qRT-PCR

Supplemental Table 1

| shRNA HELLS | GAACAAAGAAGTATCCATATT |
| --- | --- |
| shRNA BlackMamba | AGACAGATCTTGATAGAAATT |

Supplemental Table 2

| **qRTPCR** | **FORWARD** | **REVERSE** |
| --- | --- | --- |
| **BlackMamba** | TTGGAACTACTGCCGGTGTC | GTGCGAGGCTGTTTACCTCT |
| **CHMP2A** | ATGGACCTATTGTTCGGGCG | TCTCTAGTTTCTGTCGCTCGC |
| **HELLS** | AGCGGTTGTGAGGAGTTAGC | CATGCCTGGACACTCACCC |
| **PAK2** | TGAGCAGAGCAAACGCAGTA | AGGGCCATAAGCTTTCCGTG |
| **RHOU** | GCCCCTCATCCTTCCAGAAC | GAGATCCGACTGCGTTCCAA |
| **RHOA** | GGACTTAAGCGTCTGGCTC | AGTGCCACCCATGAGAACTG |
| **ECT2** | TTTTGAATCGGTTGTGGCGG | CTCTTCAAACGCCGACTCCT |
| **CDK1** | GGCTCTTGGAAATTGAGCGG | GGTATGGTAGATCCCGGCTT |
| **PLK1** | AGAAGACCCTGTGTGGGACT | ACCTCGAAACTGTGCCCTTT |
| **ANLN** | TCAGACCCAAAGGTTGAGCA | AGGACATCACTGAAGAGGTCA |
| **AURKB** | ATCAGCTGCGCAGAGAGATCGAAA | CTGCTCGTCAAATGTGCAGCTCTT |
| **PRC1** | AGCATCCTGAGTGGTGGGTA | AACTGTCAGAGAGGGACGGA |
| **KIF23** | TGGTGCAGAGTCTGAATGGAC | GCTTTTTGCGCTTGGGTTGT |
| **KIF20A** | AGTATCCCAGGAGGAGCAAGT | ATCGTCATCGGACAGCAAGC |
| **KIF4A** | AACCTTTGTTGGATGTGGGC | TGACTTAGCACCCTTCTGGAG |
| **KLHL21** | TTTGTCAGGGATGACTCCGC | CATGTACCTGATTCATGGACGG |
| **PITPNM1** | ACTACGCCAGAAGGCAATGT | ATAACCGGCCACGATGTTCA |
| **CDC42SE2** | GCGACGATAGGGCCAGATTT | TGCATACAGATGACCGCAGA |
| **TFAP2A** | ATATCCGTTCACGCCGATCC | CCTCGCAGTCCTCGTACTTG |
| **E2F1** | AGCTGGACCACCTGATGAAT | GAGGGGCTTTGATCACCATA |
| **ETS1** | TGGCCCCAGACTTTGTTGG | GCGGGATTCTGGATAGGCTG |
| **ELK1** | ATTACGACAAGCTCAGCCGG | TGTAGACGAACTTCTGGCCG |
| **FOXP3** | AACCTTCCAGGGCCGAGAT | ACCATGACTAGGGGCAGTGT |
| | **NFIC** | | --- | |  | |  | | ATGTATTCGTCCCCGCTCTG | TGAACCAGGTGTAGGCGAAG |
| **SP1** | GGTTCGCTTGCCTCGTCAG | TGATCTTGGTCGCTCATGGT |
| **MYB** | CTGGGAAGGGGACAGTCTGA | GCTGGTGCCATTAAAACGGA |
| **MYC** | TCAAGAGGCGAACACACAAC | GGCCTTTTCATTGTTTTC |
| **KLF4** | TCAACGATCTCCTGGACCTG | ATCGGATAGGTGAAGCTGCA |
| **IL-6** | CCAGAGCTGTCCAGATGAGT | GAGTTGTCATGTCCTGCAGC |
| **YY1** | CTGGAGGGCGAGTTCTCG | TCTGTTCTTCAACCACTGTCTCA |
| **MLL1** | ACCTGGAGGTAATGCTTTCAGT | ATTGCTCTGCCGCAGTTTTC |

| **ChIP promoters** | **FORWARD** | **REVERSE** |
| --- | --- | --- |
| **PAK2 promoter** | TGGCTGCTCCTCTCAATACAA | GCTGGGAGTAGTAGTTCGGTGA |
| **RHOU promoter** | GTCACCCTCTTGGGAGCTG | GAGGACCTGGAACACACGTT |
| **P4_BlackMamba** | GGCGAGGTTGTAGCAGAGAA | CTGTCCCCAGAAAGCAGGAG |
| **RHOA promoter** | ACAGCGACTTCGACTAAGCA | TGGGGTCTGTTTTGAGTGGA |
| **ANLN promoter** | TCGGTGTTTCTGGGGCATATC | CACTCCTGAATGTGACAACGC |
| **ECT2 promoter** | CTTATCTCAGAGTGCGCCGT | GGCGGATGGCCTGGATTTAT |
| **PLK1 promoter** | GGAAAGAGTACCCAGCAAGGGAG | CAGAGCCAAGAAGCCCTTACCA |
| **AURKB promoter** | CCCAACGGACCCTCTGATCT | GATTCAGTTGTTTGCGGGCG |
| **KIF20A promoter** | CGAGGTGCCCTACTTTAGGC | CGGCATTTCTGAACGCGAAC |
| **KLHL21 promoter** | CACCCCGACAAAGGAGGTAG | AAACACTGCCGAGTCATGGT |
| **PITPNM1 promoter** | CAAGGCTGGGTTCATGGGAT | AGAGAGAAAGGGCACTGCTG |
| **CDC42SE2 promoter** | CCATCTTTCGGAGCGTCCTT | CGGGTTAGGAATTGGCCTCT |
| **KLF4 promoter** | ACTCACGTTATTCGGGGCAC | ATCTTTCTCCACGTTCGCGT |
| **α-satellite** | CATAGAGGCCTGTGGTGGAA | ACGATGACTCCCAAACTGCT |
