# Supplementary figures and images for: The DNA-helicase HELLS drives ALK− ALCL proliferation by the transcriptional control of a cytokinesis-related program
Source: Cell Death Dis. 2021 Jan 27;12(1):130. doi: 10.1038/s41419-021-03425-0 (PMC7840974; doi:10.1038/s41419-021-03425-0)

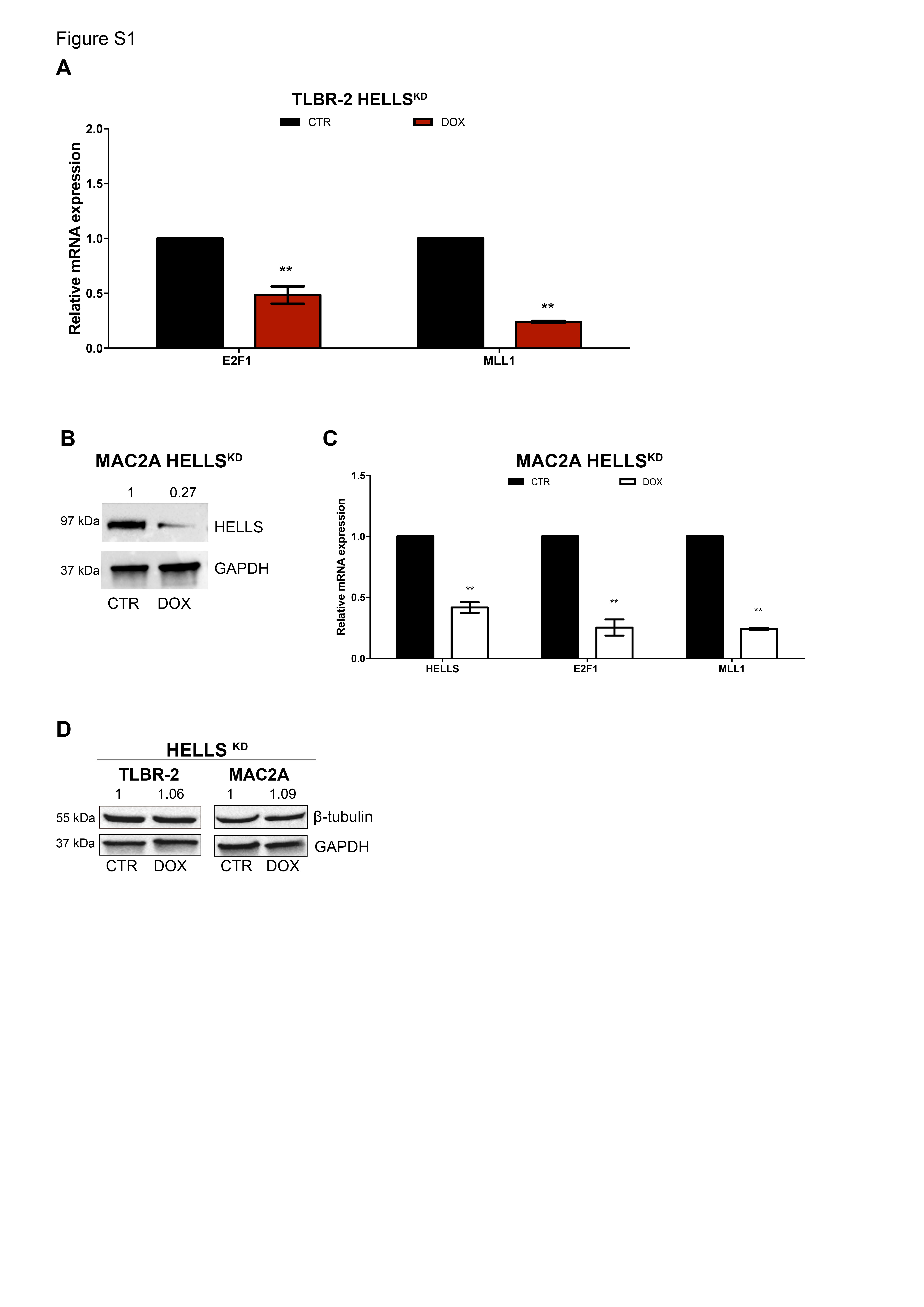

Supplement: Supplementary file 2 — Supplemental figure 1 [file 41419_2021_3425_MOESM2_ESM.jpg]

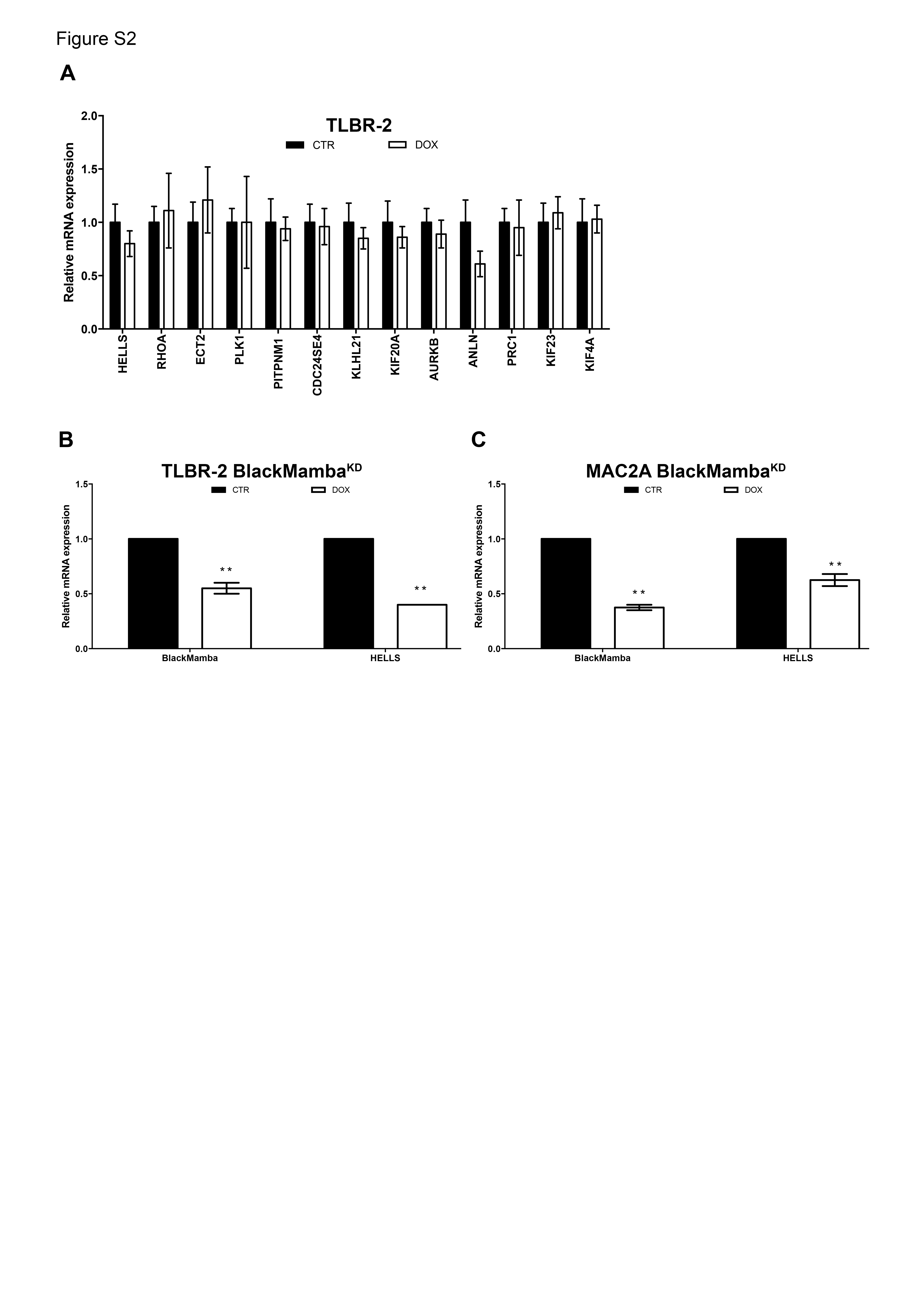

Supplement: Supplementary file 3 — Supplemental figure 2 [file 41419_2021_3425_MOESM3_ESM.jpg]

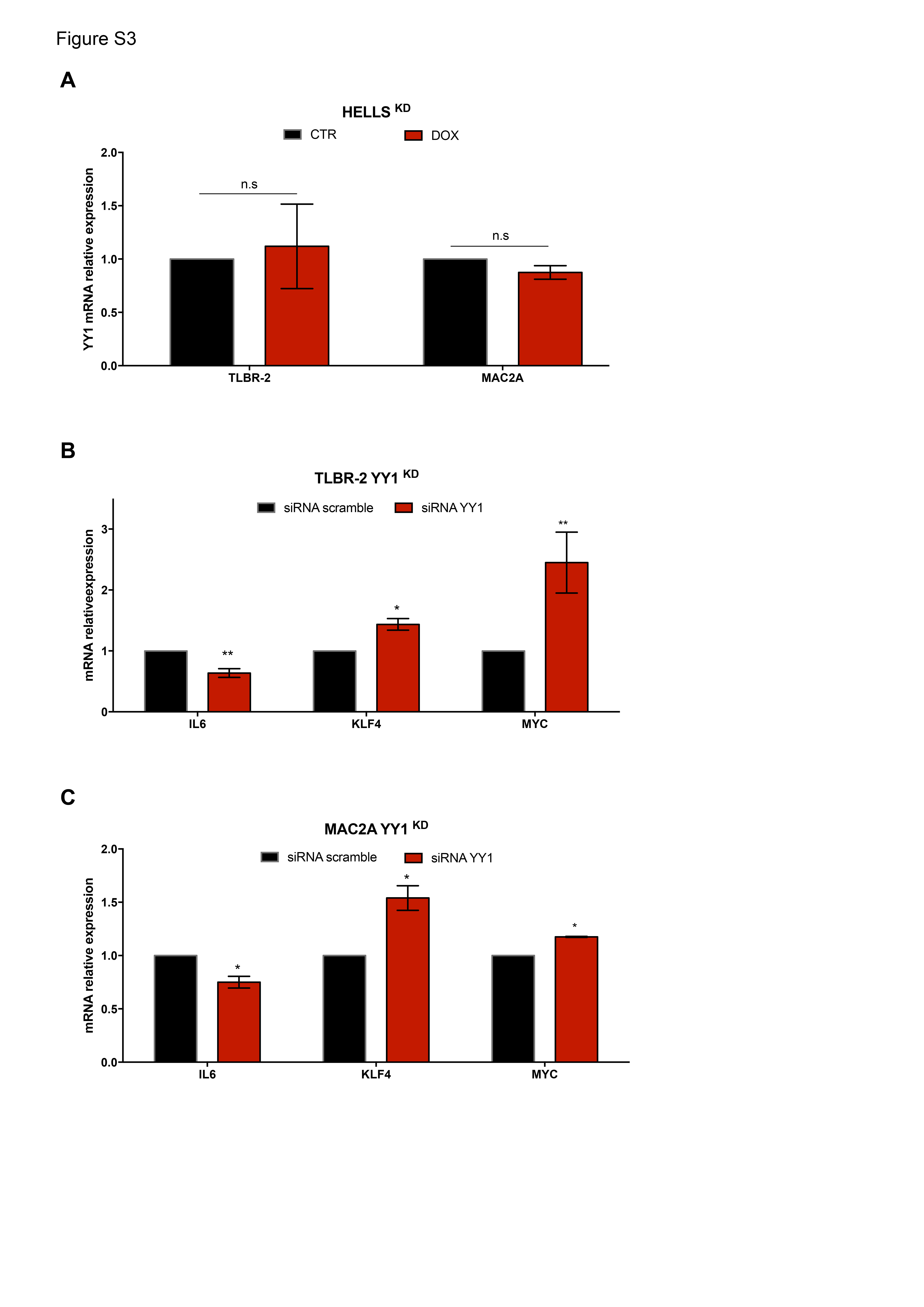

Supplement: Supplementary file 4 — Supplemental figure 3 [file 41419_2021_3425_MOESM4_ESM.jpg]
